# METTL1-driven nucleotide metabolism reprograms the immune microenvironment in hepatocellular carcinoma: a multi-omics approach for prognostic biomarker discovery

**DOI:** 10.3389/fimmu.2025.1582203

**Published:** 2025-04-22

**Authors:** Xie Weng, Yangyue Huang, Zhuoya Fu, Xingli Liu, Fuli Xie, Jiale Wang, Qiaohua Zhu, Dayong Zheng

**Affiliations:** ^1^ Department of Oncology, Shunde Hospital, Southern Medical University, The First People’s Hospital of Shunde, Foshan, China; ^2^ Hepatic Department, Integrated Hospital of Traditional Chinese Medicine, Southern Medical University, Guangzhou, China

**Keywords:** pancreatic hepatocellular carcinoma, nucleotide metabolism, non-negative matrix factorization clustering, immune cell correlation, METTL1

## Abstract

**Background:**

Hepatocellular carcinoma (HCC) remains one of the leading causes of cancer-related mortality worldwide, partly due to an incomplete understanding of the metabolic and immune dysregulation driving its progression. Here, we uncover a novel role of METTL1 in driving nucleotide metabolism reprogramming, which significantly modulates the tumor immune microenvironment.

**Methods:**

Utilizing an integrated multi-omics approach, we analyzed nucleotide metabolism-related genes derived from TCGA, GEO, and ICGC datasets. Non-negative matrix factorization (NMF) clustering stratified HCC patients into distinct subgroups with varied clinical features. Weighted Gene Co-expression Network Analysis (WGCNA) identified hub genes that were subsequently used to construct robust prognostic models via multiple machine learning algorithms. These computational findings were validated through *in vitro* experiments, immune infiltration assessments, and single-cell RNA sequencing analysis.

**Results:**

Our analyses demonstrate that METTL1 is markedly upregulated in HCC, driving a reprogramming of nucleotide metabolism that modulates the expression of key immune checkpoints, including PD-L1 and CTLA-4. This regulation is associated with an immunosuppressive tumor microenvironment, reduced infiltration of activated T cells, and poorer clinical outcomes. Moreover, the prognostic model integrating METTL1 expression and immune checkpoint profiles shows strong predictive performance across independent cohorts, highlighting its potential clinical utility.

**Conclusion:**

This study highlights the innovative role of METTL1-driven nucleotide metabolism reprogramming in reshaping the immune microenvironment of HCC. The findings provide novel insights into HCC pathogenesis and pave the way for developing personalized therapeutic strategies based on targeting METTL1 and its associated metabolic pathways.

## 1 Introduction

Liver cancer is the second leading cause of cancer-related mortality worldwide, with hepatocellular carcinoma (HCC) accounting for approximately 90% of cases (1). In 2020, nearly 900,000 new HCC cases were reported globally, highlighting its significant public health burden (2). China alone will have 410,000 new cases in 2020, accounting for 45.3% of new cases worldwide (3, 4), and is expected to cause more than 1.3 million deaths per year by 2040 (5). Common risk factors for HCC include viral infections such as hepatitis B and C, as well as chronic liver diseases (CLD) such as fatty liver, cirrhosis, alcoholic liver disease, and even metabolic diseases including diabetes, haemochromatosis, autoimmune hepatitis, and toxin exposure (1). Chronic hepatitis and cirrhosis from any other cause are the strongest risk factors for HCC, and despite advances in antiviral therapies for HBV and HCV-associated cirrhosis, the incidence of HCC continues to rise due to chronic alcohol consumption, dietary habits, and sedentary lifestyle factors (6). For HCC, tumor stage is an important prognostic factor, but less than 20% of HCC patients can be diagnosed at an early stage due to predictive power and other reasons (7), according to the Bridge to Better Outcomes in HCC (BRIDGE) study, 64% of advanced HCC cases in China are no longer eligible for radical treatment (8). The 5-year survival rate for local HCC was 32.6% and only 2.4% for metastatic HCC (9). For early HCC, tumor resection, local percutaneous ablation such as radiofrequency ablation, and liver transplantation are often used (10), however, the risk of recurrence is very high, with a 5-year recurrence rate of 40-70%, and there is no clear way to reduce the risk of disease recurrence (11); For advanced HCC, systemic therapy with sorafenib has been limited over the past decade (10). At present, the treatment of advanced HCC has been upgraded, and immune checkpoint inhibitor (ICI) has become a main treatment method in order to treat advanced HCC, and a large number of clinical studies still regard ICI as a part of the new combination therapy regimen for further research (6). However, considering the low sensitivity and drug resistance of ICI (12), we still need to continue to explore more reliable biomarkers and find key target genes, so as to improve the efficiency of immunotherapy and accuracy of prognosis prediction of HCC, and reduce its recurrence rate.

Nucleotides are the main components of cellular genetic material and exert a decisive influence on the synthesis of DNA and RNA, cell signaling, enzyme regulation and metabolism (13). Although numerous studies have documented metabolic reprogramming in HCC, the direct link between nucleotide metabolism and immune evasion remains poorly understood. In particular, most existing research has not clarified how alterations in nucleotide synthesis and degradation contribute to the modulation of immune checkpoints and the suppression of anti-tumor immunity. Notably, the role of METTL1—a key regulator of m7G tRNA methylation—in this context is largely unexplored. In this study, we aim to fill this critical gap by investigating how METTL1-driven reprogramming of nucleotide metabolism influences immune checkpoint expression and promotes an immunosuppressive microenvironment in HCC. The synthesis and excessive use of nucleotide triphosphate and its deoxidation products have been shown by a very large number of experiments to be common characteristics of cancer cells, which may play a strong role in promoting various malignant manifestations of cancer cells, such as uncontrolled proliferation, immune escape, and drug resistance, which means that the process of nucleotide metabolism may become a good entry point for tumor treatment. In addition, recent studies have shown that abnormal nucleotide metabolism may also alter the immune response of the tumor microenvironment (TME), further promoting the immune escape and drug resistance of the tumor (14). There are two synthesis pathways of purine and pyrimidine nucleotides: *de novo* synthesis and remedial synthesis. Many oncogenes have been shown to play a role in the synthesis process, such as KRAS, PI3K, MYC and other gene mutations can improve the activity of key enzymes in *de novo* pathway and promote the expression of key enzymes (15). In the process of nucleotide degradation, the inactivation or silencing of SAM domain and HD domain-containing 1 (SAMDH1) will prevent the degradation of nucleotides, thereby increasing the nucleotide content in the body (15), the occurrence and development of HCC, breast cancer, and gastric cancer can be led to by the downregulation of xanthine dehydrogenase (XDH) (16), and dihydropyrimidine dehydrogenase (DPYP), as a rate-limiting enzyme of pyrimidine degradation, plays a crucial role in the epithelial-mesenchymal transformation (EMT) of tumor cells and cancer progression (17). However, in the process of developing antitumor drugs related to inhibition of nucleotide synthesis, researchers often use analogues of nucleotide metabolites to inhibit cellular nucleotide metabolism, but this lacks specificity for cancer cells, and may inhibit normal cell metabolic processes and cause adverse reactions (13), although the research on nucleotide metabolism-related drugs for the treatment of HCC has been gradually carried out, we still need to further explore them, and find new targets for the diagnosis, treatment and prognosis of HCC.

In this study, we hypothesize that METTL1 plays a pivotal role in reprogramming nucleotide metabolism and modulating the tumor immune microenvironment in HCC. Accordingly, our objectives were to (1) evaluate the expression and prognostic significance of METTL1 in HCC (2), elucidate its impact on key metabolic pathways and immune checkpoint regulation, and (3) develop a robust prognostic model integrating multi-omics data and advanced machine learning approaches. By addressing these questions, our study aims to uncover the mechanisms underlying METTL1-mediated immune evasion and provide a foundation for the development of targeted therapeutic strategies in HCC.

## 2 Materials and methods

### 2.1 Gathering and preparation of data for analysis

To obtain bulk RNA-sequencing (Bulk RNA-seq) data from HCC patients, we downloaded the TCGA-LIHC dataset from The Cancer Genome Atlas (TCGA, https://portal.gdc.cancer.gov) using the “TCGAbiolinks” R package. In addition, we retrieved the GSE14520 and GSE76427 datasets from the Gene Expression Omnibus (GEO, https://www.ncbi.nlm.nih.gov/geo/) using the “GEOquery” R package. Furthermore, we obtained the ICGC-JP dataset from the International Cancer Genome Consortium (ICGC, https://dcc.icgc.org/) and incorporated single-cell RNA-sequencing (scRNA-seq) data from Liu et al. (Journal of Hepatology, DOI: 10.1016/j.j.Hep 2023.01.011) to enrich our study. Moreover, we downloaded metabolism-related gene sets—including those for nucleotide metabolism, amino acid metabolism, glycolysis, and lipid metabolism—from The Molecular Signatures Database (MsigDB, https://www.gsea-msigdb.org/gsea/) using the “msigdbr” R package. For all RNA-seq datasets obtained from TCGA, GEO, and ICGC, rigorous quality control (QC) procedures were applied prior to downstream analysis. Raw count data were initially processed to remove low-quality reads and samples with excessive missing values. To ensure comparability among datasets generated from different platforms and centers, batch effects were corrected using the ComBat function implemented in the sva R package. This method adjusts for technical variations while preserving the underlying biological signal. Furthermore, normalization was performed using the TMM (trimmed mean of M-values) method from the edgeR package to standardize library sizes across samples. These combined QC and batch-effect correction steps were essential for constructing an integrated and reliable dataset for subsequent analyses.

### 2.2 Molecular typing and correlation analysis

Based on nucleotide metabolism genes, we used Non-negative Matrix Factorization (NMF) algorithm to perform dimensionality reduction clustering on TCGA-LIHC. And generate line charts of cophenetic, dispersion, evar, residuals, residual sum of squares (RSS), silhouses, sparseness as the rank value changes from 2 to 10. The above line chart is comprehensively analyzed to determine the selection of the best rank value. To determine the optimal number of clusters (k) in our NMF analysis, we evaluated multiple quantitative metrics across k values ranging from 2 to 10. In particular, we assessed the cophenetic correlation coefficient, dispersion, residual sum of squares (RSS), and silhouette values. We observed that at k=2, the cophenetic correlation reached a local maximum, and the consensus matrix exhibited clear, well-defined block-diagonal structures, indicating high cluster stability. Additionally, the silhouette scores for k=2 were significantly higher compared to other k values, reflecting robust separation between the clusters. Based on these specific indicators, we selected k=2 as the optimal number of clusters for further analysis.

The matrix heat map was used to visualize the optimal partitioning results. Next, we evaluated and compared the clinical characteristics of patients across different nucleotide metabolic subsets. Specifically, we performed Kaplan-Meier (KM) survival analysis to demonstrate the differences in prognosis among the patient clusters using survival curves. Differences in overall age among the clusters were compared and visualized with box plots, and the distribution of tumor stages (stage I-IV) was presented using bar plots.

To analyze the crosstalk between nucleotide metabolism subsets and the three classical metabolic pathways (glycolysis, amino acid metabolism, and lipid metabolism), we identified metabolism-related genes that were significantly differentially expressed between the nucleotide metabolism subsets. Heat maps were generated to visualize the gene expression patterns. Additionally, we explored differences in functional pathways between the nucleotide subsets using Gene Set Enrichment Analysis (GSEA) to identify pathways that were upregulated or downregulated in each subset.

### 2.3 Weighted gene co-expression network analysis and multi-machine learning model building (prognostic model construction)

#### 2.3.1 Model building via WGCNA and machine learning

We used the “WGCNA” R package to perform weighted gene co-expression network analysis. First, we preprocessed the data to exclude genes expressed at low levels or with minimal variation across samples, ensuring a robust scale-free network. Next, we constructed a correlation matrix and applied a power function to convert it into an adjacency matrix. The optimal soft threshold (β) was determined based on two criteria (1): achieving scale-free topology characteristics and (2) maintaining appropriate connectivity or sparsity. We visualized the trend of the scale-free topology model fit and average connectivity as the soft threshold changed, and selected the best threshold accordingly. Based on this, we constructed the Topological Overlap Matrix (TOM). Subsequently, we built a gene co-expression network and employed the Dynamic Tree Cut algorithm to partition the network into distinct modules, generating corresponding cluster trees. We then analyzed the correlation between these modules and clinically relevant indicators (age, stage, survival status, OS time) as well as nucleotide metabolism subsets, with the results visualized in a module-feature relationship heat map. Hub genes were extracted from the modules most strongly associated with nucleotide metabolism subsets based on gene significance (GS) > 0.4 and module membership (MM) > 0.6. Finally, we performed Gene Ontology (GO) enrichment analysis on the genes within the co-expression modules and visualized the top five GO terms for each module using bar charts.

#### 2.3.2 Prognostic model construction and model validation

A prognostic prediction model was constructed based on the hub genes identified above. We employed the “Mime” package to integrate 10 different algorithms, resulting in 101 machine learning model combinations. Using TCGA-LIHC (Dataset1) as the training set and GSE14520 (Dataset2), GSE76427 (Dataset3), and ICGC-JP (Dataset4) as validation sets, we input the hub genes into the machine learning pipeline, calculated the C-index for all four datasets, and selected the combination with the highest average C-index across all cohorts as the final model. The predictive performance of this model was then verified using multiple datasets. We divided the datasets into two risk groups based on the median risk score and compared their prognoses using Kaplan-Meier survival curves. Time-dependent Receiver Operating Characteristic (ROC) curves were generated using the 1-, 3-, and 5-year survival rates; an area under the curve (AUC) > 0.6 was considered indicative of good predictive performance. Finally, we performed a meta-analysis of univariate Cox regression to comprehensively assess the efficacy of the prognostic model. To ensure the robustness and generalizability of our prognostic model, we employed a 5-fold cross-validation strategy for each dataset. In this approach, the dataset was randomly divided into five equal parts. In each iteration, four parts were used for training and the remaining part was used for testing. This process was repeated until each fold had served as the test set exactly once. Performance metrics, including the concordance index (C-index) and time-dependent ROC values, were then averaged across the five folds. This rigorous cross-validation procedure minimized potential overfitting and validated the model’s predictive power across the TCGA, GEO, and ICGC cohorts. Moreover, the same procedure was applied within our integrated machine learning pipeline to compare and select the optimal candidate model based on the highest average C-index.

### 2.4 Differences in expression profiles of risk groups

By generating a Sankey diagram, we visualized both the distribution of the two risk groups across the different nucleotide metabolic subsets and the distribution of risk groups according to patient survival (Alive and Death). Next, we analyzed the differences in gene expression between the risk groups. Genes with log_2_FoldChange > 0.5 and adjusted P-value < 0.05 were considered significantly differentially expressed, and these DEGs were visualized using a volcano plot. Additionally, we compared and visualized the differences in the activity of classical cancer-related pathways and model gene expression levels between the risk groups using heat maps. We also employed a butterfly plot to illustrate the differences in the activity of apoptosis-related and proliferation-related genes between the risk groups. Moreover, we analyzed the mutations in the two groups by generating waterfall plots to display the top 10 genes with the highest mutation rates, as well as the mutation types (single nucleotide polymorphisms) for these genes. Finally, we calculated enrichment scores for 13 classical tumor pathways and analyzed their correlations with risk scores, with the results visualized in correlation heat maps.

### 2.5 Analysis of the correlation between risk score and immune microenvironment

We used the “IOBR” R package, which incorporates several TME parsing algorithms (CIBERSORT, EPIC, MCP, quanTIseq, TIMER, and xCell), to analyze immune infiltration in the TCGA-LIHC dataset. We quantified the relative abundance of 22 immune cell types in the two defined risk groups and visualized the results with box plots. We also examined the correlation between immune cell infiltration and the risk score using these six algorithms. In addition, the ESTIMATE algorithm was applied to evaluate the relative levels of immune and stromal components in different risk groups, with ImmuneScore and StromalScore differences visualized using box plots. Furthermore, immunophenotype scores (IPS) for TCGA-LIHC samples, downloaded from the TCIA database (https://tcia.at/home), were used to predict patient responses to immune checkpoint inhibitors (anti-CTLA-4 and anti-PD-1). IPS were categorized into four types: ips_ctla4_pos_pd1_pos, ips_ctla4_pos_pd1_neg, ips_ctla4_neg_pd1_neg, and ips_ctla4_neg_pd1_pos. The differences in IPS between the two groups are displayed in the figure.

### 2.6 Single cell analysis

We downloaded single-cell data from patients after ICB treatment from the Journal of Hepatology and performed a single-cell analysis. The UMAP algorithm was used to reduce the high-dimensional single-cell data to two dimensions, allowing us to identify multiple cell clusters under a specific resolution. UMAP visualizes the distribution of these clusters and highlights differences between two ICB response groups: non-response (NR) and response (R). Using cell annotation information from the literature, we classified cells into several major cell types, and their distribution was visualized on the UMAP map. Next, we compared the NR and R groups and found differences in the composition and proportion of cell types. We also used the AddModuleScore function in the “Seurat” package to score the expression levels of model genes in different treatment response groups. The distribution of the signature score was shown on the UMAP, and a box plot was used to compare the overall signature score between the two groups. Subsequently, we ranked the different cell types in descending order according to the signature positive ratio, and the results were displayed in a bar chart. To specifically analyze the activity differences of the model genes between NR and R groups, we generated heat maps to display their expression profiles. Finally, we divided all cells into high and low score groups based on the median signature score, used GSEA to identify functional pathways that were significantly upregulated or downregulated in the high-score group, and plotted bubble maps accordingly.

### 2.7 Correlation analysis of single gene METTL1 and immune landscape

We analyzed the effects of single-gene METTL1 on the immune landscape across multiple cancer types. First, we calculated the correlation coefficients between METTL1 and five types of immunoregulatory factors (receptors, chemokines, immunoinhibitors, immunostimulators, and MHC molecules) in 33 cancer types and visualized the results in a heat map. Next, we performed a multi-cancer analysis to examine the correlations between METTL1 and four immune checkpoints—CD274 (PD-L1), CTLA-4, LAG-3, and PDCD1 (PD-1)—using Pearson correlation (r) as the metric. In addition, we investigated the relationship between METTL1 and the infiltration levels of 28 immune cell subtypes, quantifying immune cell abundance with the ssGSEA method and presenting the correlations in heat maps.

Furthermore, we specifically analyzed the association of METTL1 with various immune features in Liver Hepatocellular Carcinoma (LIHC). First, patients were divided into high and low METTL1 expression groups, and the activity levels of the five types of immunomodulators were compared between these groups using heat maps. We then compared the degree of gene enrichment at each stage of the anticancer immunity cycle between the two METTL1 status groups, with the differences clearly visualized in box plots. Next, we quantified and visualized the expression levels of effectors associated with five tumor-infiltrating immune cell types (dendritic cells, CD8+ T cells, macrophages, Th1 cells, and NK cells) in these subgroups. Finally, we analyzed the correlation between METTL1 and 24 immunosuppressive molecules, and the results were visualized using a correlation pie chart.

### 2.8 Cell culture and RT-qPCR

Human hepatocellular carcinoma cell lines (HepG2, Hep3B, Huh-7) and a normal human liver cell line (LX2) were purchased from ATCC (Manassas, VA, USA) and cultured in DMEM medium (Solarbio, Beijing, China) supplemented with 10% FBS and 1% penicillin-streptomycin. Total RNA was isolated using TRIzol reagent (Invitrogen, Carlsbad, CA, USA), and genomic DNA was removed using the gDNA Remover kit. Subsequently, RNA was reverse-transcribed into cDNA using the ReverTra Ace qPCR RT Master Mix. Real-time quantitative PCR (qPCR) was performed using SYBR Premix Ex Taq II on the Mx3005P real-time fluorescence quantitative PCR system (Stratagene, San Diego, CA, USA). GAPDH was used as an endogenous control for mRNA normalization. The reaction conditions were as follows: initial denaturation at 95°C for 10 minutes, followed by 45 cycles of denaturation at 95°C for 5 seconds and annealing at 60°C for 30 seconds. Amplification of target genes and the internal reference gene was performed separately for each sample, with three replicate wells per sample group. Data were analyzed using the 2^-ΔΔCt method. The primer sequences are shown in **Table 1**.

**Table 1:** Primer sequences used for qPCR analysis (5’ → 3’).

Gene	Primer Direction	Sequence (5’ → 3’)
GAPDH	Forward	GGAGCGAGATCCCTCCAAAAT
GAPDH	Reverse	GGCTGTTGTCATACTTCTCATGG
METTL1	Forward	GGCAACGTGCTCACTCCAA
METTL1	Reverse	CACAGCCTATGTCTGCAAACT

### 2.9 Statistical analysis

Our statistical analysis is based on the software R.4.3.1. Unless otherwise stated, the production of pictures is achieved by the “ggplot” software package. The KM survival curve was used to analyze survival, as well as we evaluated the ROC curve using the AUC. The p value was used to determine whether the difference was statistically significant, *: p < 0.05; **: p < 0.01; ***: p < 0.001.

## 3 Results

### 3.1 Typing analysis and correlation assessment

**Figure 1:**
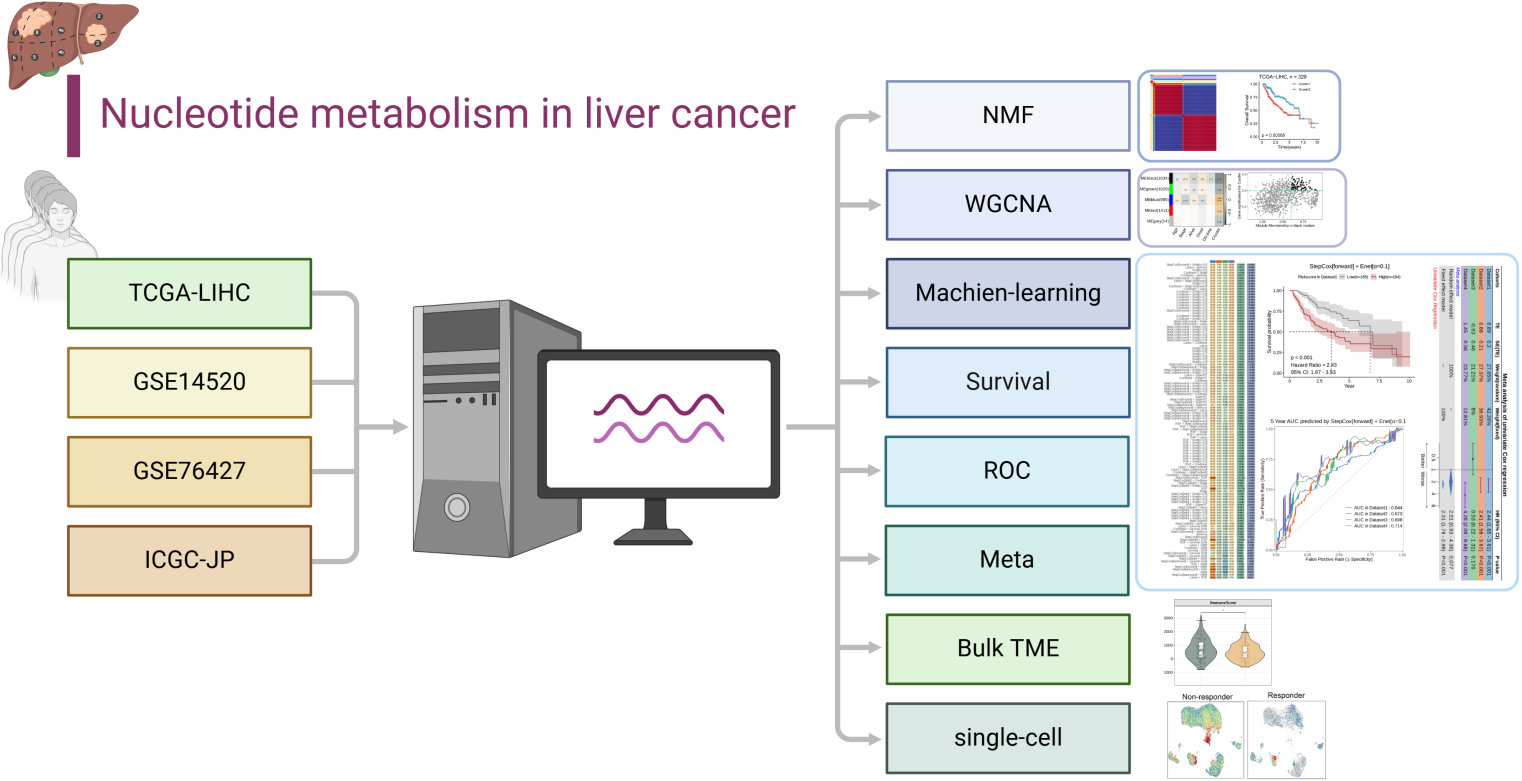
Workflow of the study.

The workflow for this article is shown in **Figure 1**.

**Figure 2:**
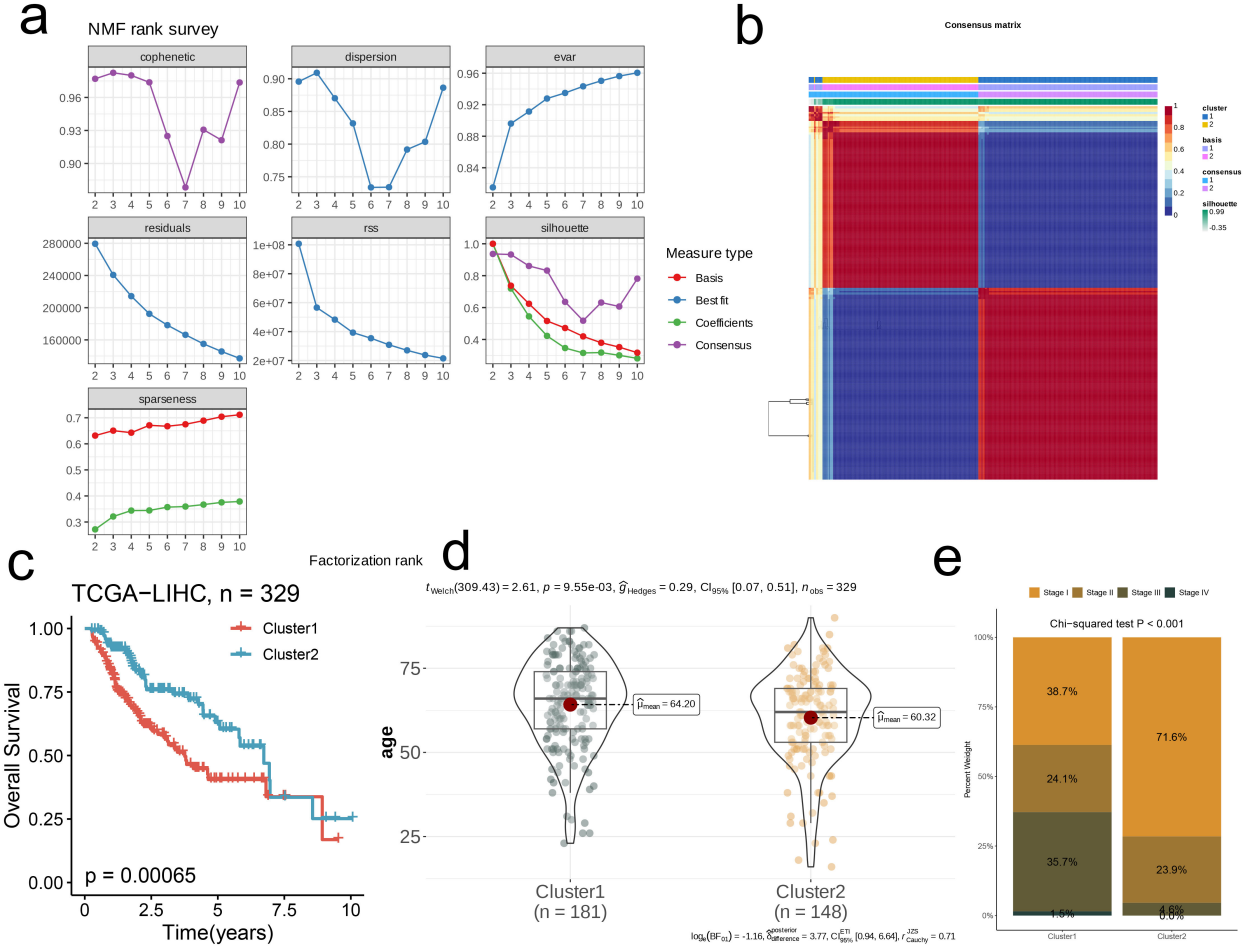
Nucleotide metabolism subclusters and prognosis in TCGA-LIHC. **(a)** Cophenetic distributions, residual sum of squares (RSS), and dispersion indices for ranks 2–10. **(b)** Consensus map from non-negative matrix factorization clustering (K = 2). **(c)** Overall Kaplan-Meier survival curves for both subclusters. **(d)** The age distribution between two subclusters. **(e)** The stage distribution between two subclusters.

First, we successfully reduced the dimension of TCGA-LIHC by NMF algorithm. Comprehensive evaluation criteria of cophenetic, dispersion, evar, residuals, residual sum of squares (RSS), silhouses, sparseness, We obtained the optimal rank value to achieve molecular typing (**Figure 2a**). At the same time, based on the principle of “high cohesion, low coupling”, we selected k=2 to obtain the consistent score matrix of all samples, and successfully obtained two nucleotide metabolic subgroups C1 and C2. This further demonstrates the powerful power of NMF clustering (**Figure 2b**). The survival difference within the two subpopulations is shown by the KM survival curve, with C1 having a worse prognosis (p=0.00065, **Figure 2c**). Subsequently, we compared and analyzed the overall age difference between the two subpopulations, and found that the overall age of C1 was significantly higher than that of C2 (p<0.05, **Figure 2d**). The bar chart’s results showed that the clinical stage of C1 was biased towards late stage, while that of C2 group was mainly concentrated in early stage, which confirmed the results of survival analysis (p<0.001, **Figure 2e**).

**Figure 3:**
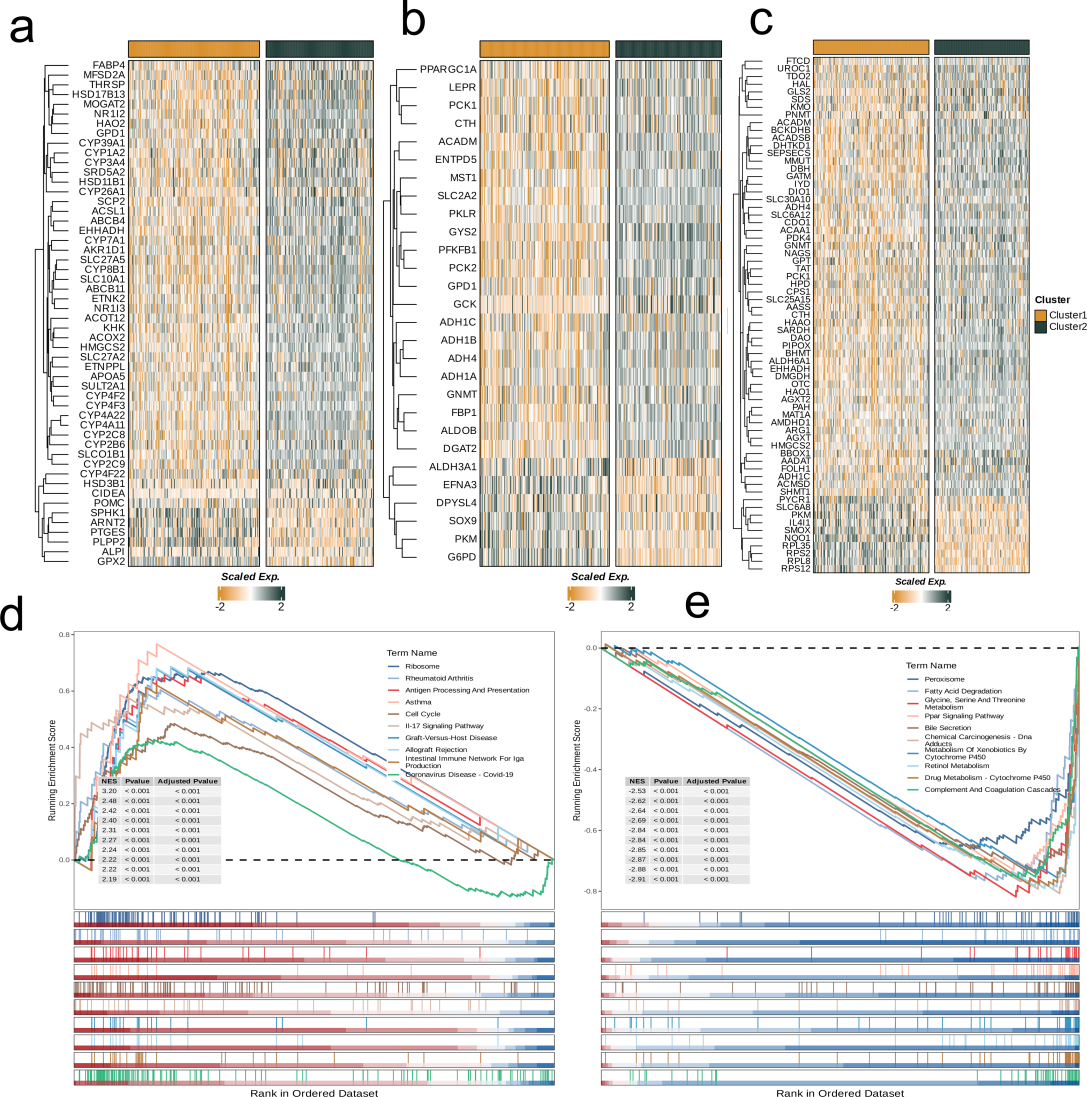
Crosstalk between nucleotide metabolism subclusters and key metabolic pathways. **(a)** Differences in glycolysis-related genes between subclusters. **(b)** Differences in amino acid metabolism-related genes between subclusters. **(c)** Differences in lipid metabolism-related genes between subclusters. **(d)** Gene set enrichment analysis (GSEA) reveals pathways downregulated in subtype C1 relative to C2. **(e)** GSEA reveals pathways upregulated in subtype C1 relative to C2.

Next, to explore the two subgroups’ metabolic characteristics and differences, we selected three classical metabolic pathways (glycolysis, amino acid metabolism, and lipid metabolism) and compared the metabolism-related genes with significant differences in expression between different subgroups and visualized them with heat maps. It was observed that the gene expression levels’ distribution in these three metabolic pathways of those two subpopulations was similar, and most of the genes related to C1 showed relatively low expression (**Figures 3a–c**). In addition, we used GSEA to assess the differential pathways between the two subpopulations. All these results showed that C1 was down-regulated in Ribosome, Antigen Processing And Presentation, Cell Cycle, IL-17 Signaling Pathway and other pathways. In Peroxisome, Glycine, Fatty Acid Degradation, Ppar Signaling Pathway, Serine And Threonine Metabolism, Bile Secretion, Chemical carcinogenes-DNA Adducts were up-regulated (p<0.001, **Figures 3d, e**).

### 3.2 Model construction and model verification based on WGCNA

**Figure 4:**
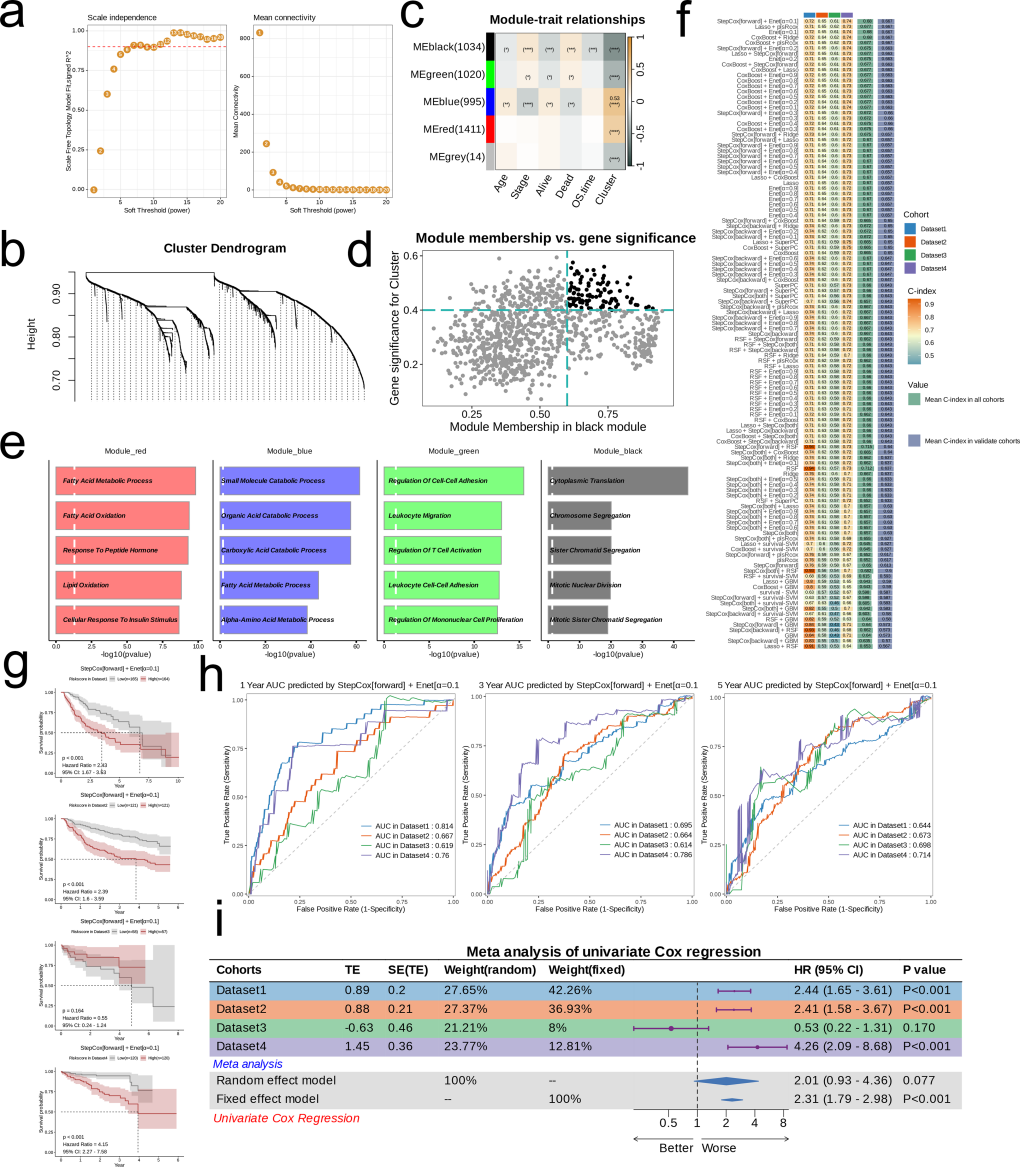
Models Construction based on nucleotide metabolism subclusters. **(a)** Analysis of network topology for different soft-threshold power. The left panel shows the impact of soft-threshold power (power = 7) on the scale-free topology fit index; the right panel displays the impact of soft-threshold power on the mean connectivity. **(b)** Cluster dendrogram of the coexpression modules. Each color indicates a co-expression module. **(c)** Module-trait heatmap displaying the correlation between module eigengenes and clinical traits. **(d)** Correlation between module membership and gene significance in the black module. Dots in color were regarded as the hub genes of the corresponding module (MM > 0.6 & GS > 0.4). **(e)** Top five enriched GO terms of module genes in each module except for the grey. **(f)** A total of 101 kinds of prediction models fitted in TCGA-LIHC (Dataset1) and verified in the other three validation cohorts (GSE14520 [Dataset2], GSE76427 [Dataset3], and ICGC-JP [Dataset 4]). The model was ordered by the average of the C-index of validation datasets. The optimal model developed by “StepCox[forward] + Enet[α=0.1]” was utilized in subsequent analyses. **(g)** Survival differences between two groups in the four datasets. **(h)** Time-dependent ROC analysis of the model in the four datasets. **(i)** Meta analysis of univariate Cox regression across the four datasets.

We applied WGCNA to identify key gene modules associated with nucleotide metabolism and clinical indicators. The optimal soft threshold (β = 7) was chosen based on scale-free topology and moderate connectivity (**Figure 4a**). We made a cluster tree diagram of the co-representation module to show the clustering levels and effects (**Figure 4b**). Consensus clustering identified five modules, with the MEblack module showing a significant correlation with clinical features (age, stage, survival status, OS time) and nucleotide metabolism subsets (p<0.05; **Figure 4c**). Hub genes from the MEblack module were then selected for further analysis (**Figure 4d**), and GO enrichment analysis revealed that each module was enriched in distinct biological pathways—for example, Module_red in lipid metabolism, Module_blue in small molecule catabolism, Module_green in immune-related processes, and Module_black in cell division (**Figure 4e**).

Using these hub genes, we developed 101 prognostic models by integrating 10 machine learning algorithms with TCGA-LIHC as the training set and GSE14520, GSE76427, and ICGC-JP as validation sets. The “StepCox[forward] + Enet[α=0.1]” model, which achieved the highest average C-index, was selected as the final prognostic model (**Figure 4f**). Patients were stratified into high- and low-risk groups based on the median RiskScore. Kaplan-Meier survival curves demonstrated significantly lower survival in the high-risk group in TCGA-LIHC, GSE14520, and ICGC-JP (p<0.001; **Figure 4g**), while ROC curves confirmed good predictive efficiency (AUC > 0.6 for 1-, 3-, and 5-year survival; **Figure 4h**). A meta-analysis of univariate Cox regression further supported the model’s robust prognostic performance (**Figure 4i**).

### 3.3 Differences in expression profiles of risk groups

**Figure 5:**
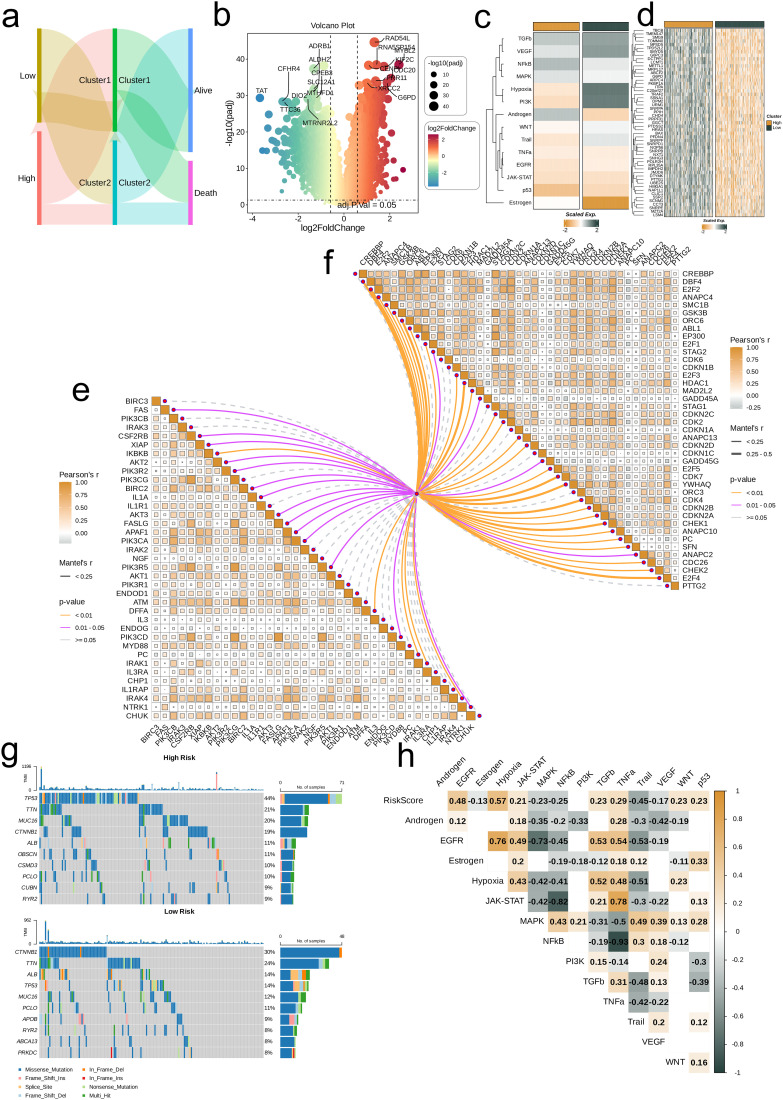
Associations between risk scores, clinical features, and oncogenic pathways in TCGA-LIHC. **(a)** Distribution of risk groups among nucleotide metabolism subclusters and survival samples. **(b)** Differential genes between risk groups. **(c)** Activity differences in classic cancer-related pathways between risk groups. **(d)** Relationships between risk groups and model gene expression levels. **(e)** Correlation of risk scores with apoptosis-related genes. **(f)** Correlation of risk scores with cell proliferation-related genes. **(g)** Distribution of the top 10 genes with the highest mutation frequencies across different risk groups. **(h)** Correlation of risk scores with enrichment scores of different classic tumor pathways.

The distribution of high- and low-risk groups across different nucleotide metabolism subsets and survival samples (Alive and Death) was visualized using Sankey diagrams. It is not difficult to find that most of the low-risk groups are distributed in C2, and only a few of C2 are dead samples. Sankey diagrams revealed that most low-risk patients are distributed in nucleotide metabolism subset C2—with few dead samples—whereas high-risk patients predominantly belong to C1 and exhibit a worse prognosis (**Figure 5a**). Differential expression analysis showed that genes such as RAD54L, MYBL2, and CDC20 are significantly overexpressed in the high-risk group, while ADRB1, ALDH2, and CFHR4 are downregulated (**Figure 5b**). Furthermore, classical cancer-related pathways, notably Hypoxia and PI3K, display higher activity in the low-risk group (**Figure 5c**), and heat maps confirmed that model gene expression is generally elevated in the high-risk group (**Figure 5d**). The butterfly diagram further illustrated the correlations between risk scores and genes involved in cell proliferation and apoptosis (**Figures 5e, f**). Mutation analysis indicated that TTN is commonly mutated in both groups, with TP53 mutations being more prevalent in the high-risk group (**Figure 5g**). Finally, correlation heat maps revealed a significant negative correlation between risk scores and the NFκB/TNF-α pathways, and a strong positive correlation between EGFR and pathways such as Hypoxia, JAK-STAT, and TNF-α (**Figure 5h**).

### 3.4 Correlation analysis between risk score and immune microenvironment

**Figure 6:**
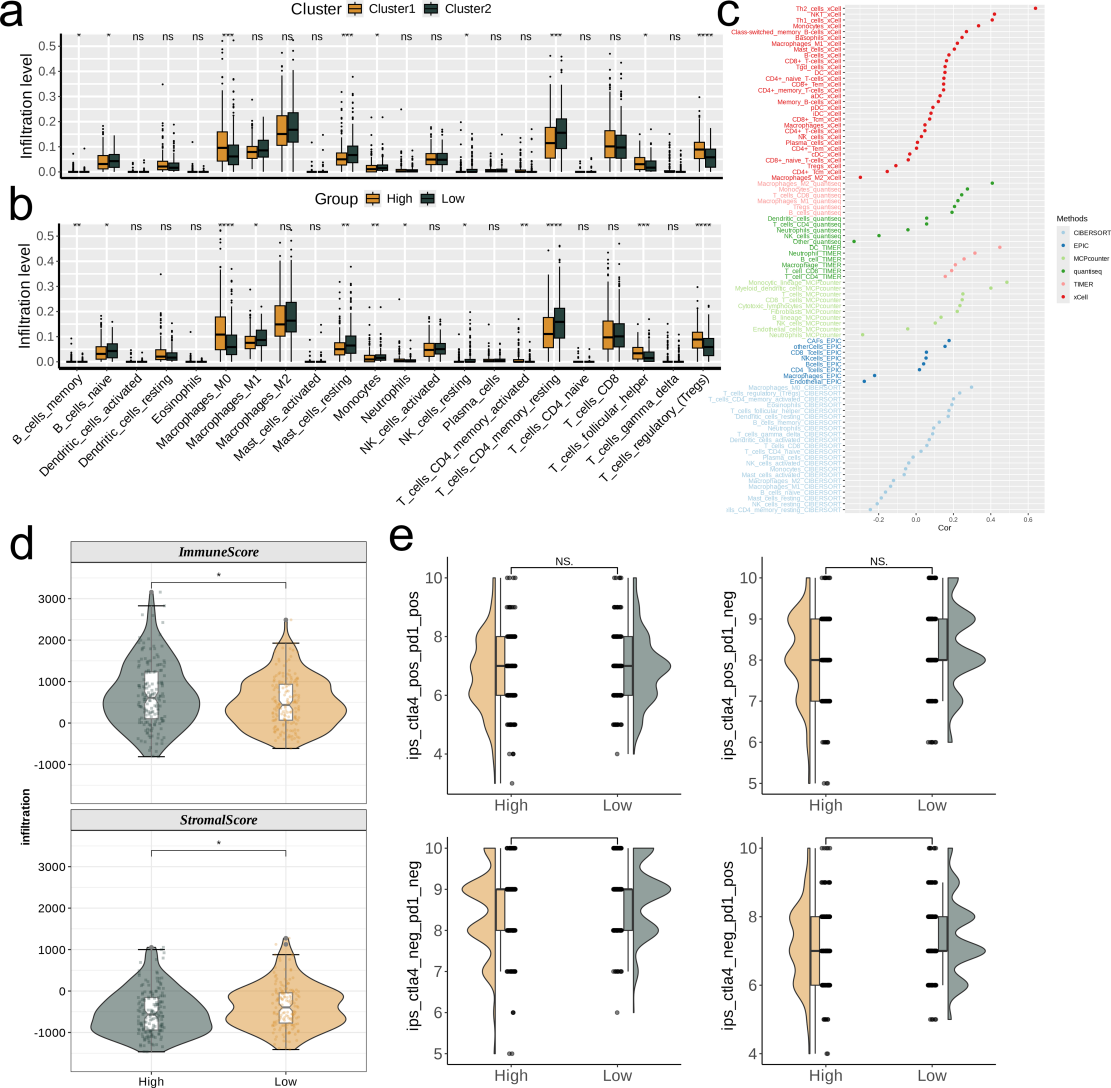
Investigation of the correlation between risk score and the immune microenvironment of TCGA-LIHC. **(a, b)** Differences in infiltration levels of 22 immune cell types between nucleotide metabolism subclusters and between risk groups. **(c)** Correlation of risk scores with various immune cells as revealed by six different algorithms. **(d)** Differences in tumor microenvironment scores between different risk groups as revealed by the ESTIMATE algorithm. **(e)** Differences in IPS scores predicting effectiveness of PD-L1 or CTLA-4 inhibitor treatments between different risk groups. IPS score of each TCGA-LIHC sample was acquired from the TCIA (https://tcia.at/home) In the figure, “*”, “**”, and “***” indicate p < 0.05, 0.01, and 0.001 respectively, while NS denotes no significant difference.

The boxplot shows that among all statistically significant scoring items, for the two nucleotide metabolism subgroups, the infiltration levels of Macrophages_M0, T_cells_follicular_helper, and T_cells_regulatory_(Tregs) are higher in subgroup C1 compared to subgroup C2. For all other immune cell infiltrations, the levels are significantly higher in subgroup C2 than in subgroup C1 (p<0.05). Furthermore, the differences in immune cell infiltration levels vary between different risk groups. Specifically, the infiltration level of Macrophages_M0 is significantly higher in the high-risk group compared to the low-risk group, while the infiltration level of T_cells_CD4_memory_resting is significantly lower in the high-risk group compared to the low-risk group (p<0.05, **Figures 6a, b**). In addition, we explored the correlation of risk scores with various immune cells using six different algorithms (**Figure 6c**). By quantifying scores, we compared the relative levels of immune and matrix components between different risk groups. It can be found that the ImmuneScore of the high-risk group was higher than that of the low-risk group and the StromalScore was lower than that of the low-risk group (p<0.05, **Figure 6d**). According to the nimbus map, based on the IPS differences in the treatment effectiveness of CTLA-4 and PD-1 inhibitors, we found that the IPS scores of the low-risk group were higher than those of the high-risk group (**Figure 6e**).

### 3.5 Single cell analysis

**Figure 7:**
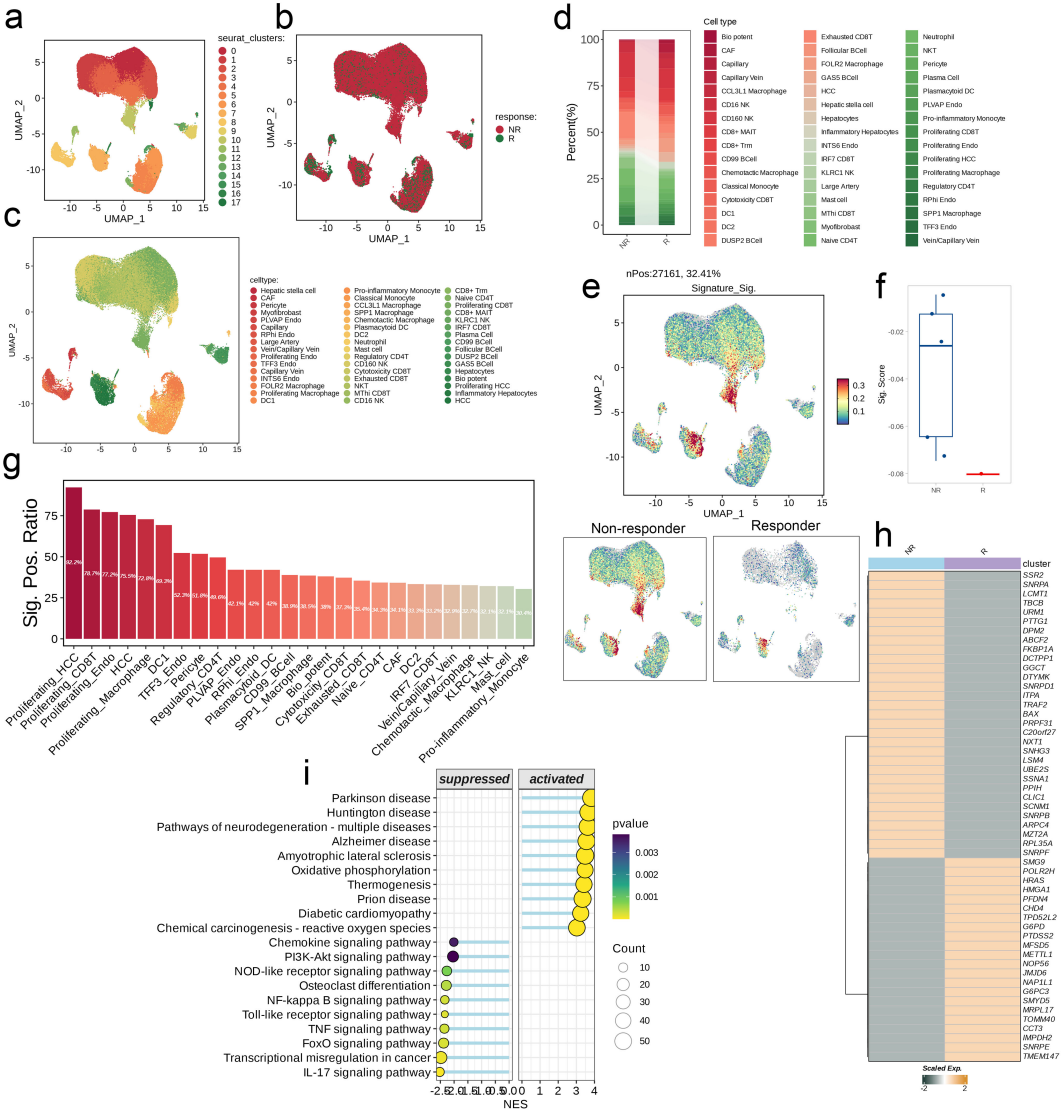
Single-cell analysis of risk score in immunochemotherapy treated scRNA-seq cohort. **(a–c)** UMAP visualization the public liver cancer scRNA-seq cohort treated with immunochemotherapy. **(d)** Differences in the abundance of cell types across different groups. **(e)** Distribution of the signature scores between groups. The signature score was calculated by the AddModuleScore() function implemented in the Seurat package based on the genes derived from the model from the machine-learning pipeline. **(f)** The signature scores of each patient between two different groups. **(g)** The positive ratio of the signature across each cell type. **(h)** The differences in the abundance of signature genes between different groups in all patients. **(i)** GSEA reveals significantly altered pathways in cells with high signature scores compared to those with low scores.

By using UMAP dimensional-reduction clustering algorithm, immunochemotherapy treated scRNA-seq cohort was divided into multiple clusters (**Figures 7a–c**). Subsequently, the comparison of cell type abundance between the NR group and the R group showed that the proportion of Exhausted CD8T cell subsets in the NR group was significantly higher than that in the R group, while Vein/Capillary Vein accounted for a very small proportion in both groups (**Figure 7d**). Then, AddModuleScore was used to score each cell. We not only showed the distribution of signature score with the UMAP, but also combined with the boxplot results. It was found that the score of the NR group was significantly higher than that of the R group (**Figures 7e, f**). In addition, we also found that the signature positive ratio varies among cell types, Proliferating_HCC cell subgroup is the highest (92.2%) and the Pro-inflammatory_Monocyte cell subgroup is the lowest (30.4%, **Figure 7g**). Heat map results showed that there were significant differences in the distribution of model gene scores between the NR group and the R group (**Figure 7h**). Finally, we divided all cells into high-low groups based on the median score and used GSEA to assess the difference pathways between the two groups. It was found that the high-score group was up-regulated in Parkinson disease, Huntington disease, Pathways of neurodegeneration-multiple diseases, etc. Down-regulated in Chemokine signaling pathway, PI3K-Akt signaling pathway, NOD-like receptor signaling pathway and other pathways (**Figure 7i**).

### 3.6 Correlation analysis of single gene METTL1 and immune landscape

**Figure 8:**
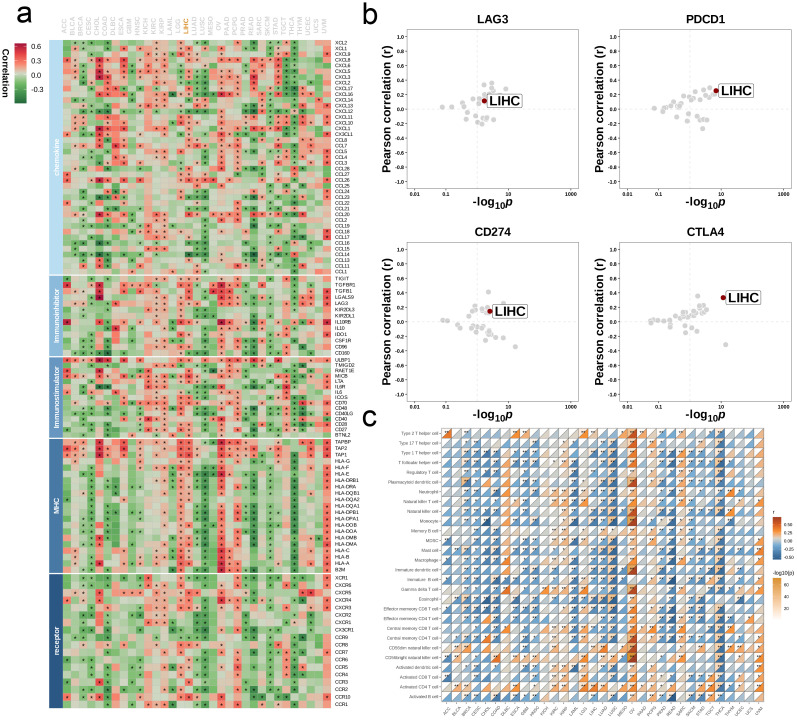
Influence of METTL1 on immune landscapes in pan-cancer. **(a)** Association of METTL1 with various immunoregulators (including receptors, MHC molecules, immunostimulators, and chemokines). **(b)** The associations between METTL1 and four immune checkpoints: CD274 (PD-L1), CTLA-4, LAG-3, and PDCD1 (PD-1), with dots representing various cancer types. **(c)** Relationship of METTL1 with infiltration levels of 28 tumor-associated immune cells, as analyzed by the ssGSEA method. The correlation strength is depicted by color intensity. Statistically significant correlations, determined through Pearson correlation analysis, are marked with asterisks. *P < 0.05; **P < 0.01.

We analyzed the single gene METTL1 at the pan-cancer level to assess its predictive power and biological significance in multiple cancers. We first calculated the correlation coefficients between METTL1 and 5 types of immunoregulatory factors (receptors, MHC, immunostimulator, immunoinhibitor, chemokine) in 33 types of cancer. It was found that most of the immunomodulators in LIHC were strongly correlated with METTL1 (**Figure 8a**). Then, we conducted a multi-cancer analysis on the correlation between the four immune checkpoints CD274 (PD-L1), CTLA-4, LAG-3, and PDCD1 (PD-1) and METTL1, and LIHC also showed a strong correlation with them (**Figure 8b**). By using ssGSEA to quantitatively score the infiltration levels of 28 tumor-associated immune cells, we also explored the correlation between 33 cancers and them (**Figure 8c**). These results all reveal the immunological relevance of METTL1 at the pan-cancer level.

**Figure 9:**
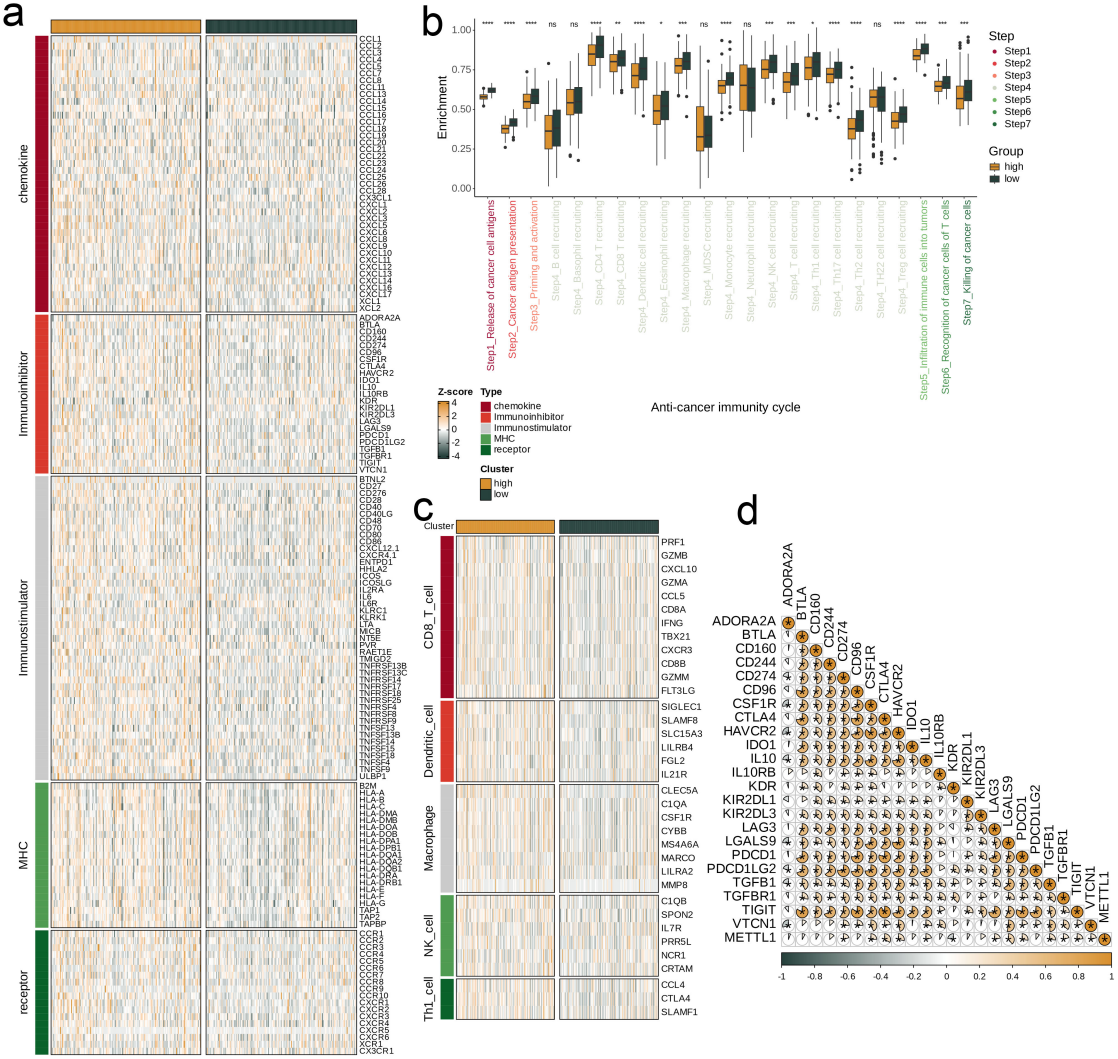
Impact of METTL1 on the TME in TCGA-LIHC. **(a)** Expression differences of immunoregulators (as identified in **Figure 8a**) between the high- and low-METTL1 expression groups in TCGA-LIHC. **(b)** Variations in the stages of the cancer immunity cycle for high versus low METTL1 expression groups. **(c)** Association of METTL1 with the expression of effector genes of five types of tumor-infiltrating immune cells: CD8+ T cells, DCs, macrophages, NK cells, and Th1 cells, determined by the six TME decoding algorithms. **(d)** Expression differences in effector genes of these immune cells between the high- and low- METTL1 groups. Asterisks denote the significance levels as determined by the Mann-Whitney U test. ns, not significant; *P < 0.05; **P < 0.01; ***P < 0.001; ****P < 0.0001.

Next, our research focuses on exploring the relationship between METTL1 and different immune characteristics in the context of LIHC. First, we divided the TCGA-LIHC dataset into high and low groups based on the expression level of METTL1. By observing the heat map, it can be found that the expression of immune modulator molecules is significantly different between the two groups (**Figure 9a**). Then, we evaluated the gene enrichment degree at each stage of the anti-cancer immunity cycle in the high and low expression groups, and found that the low expression group was significantly higher than the high expression group at any stage (p<0.05, **Figure 9b**). We also used heat maps to show the expression difference of related effector in 5 tumor-infiltrating immune cells (CD8_T_cell, Dendritic_cell, Macrophage, NK_cell, Th1_cell) between the two groups. It was found that the low expression group was relatively generally lower than the high expression group (**Figure 9c**). Finally, we analyzed the correlation between METTL1 and 24 immunosuppressive molecules (**Figure 9d**).

**Figure 10:**
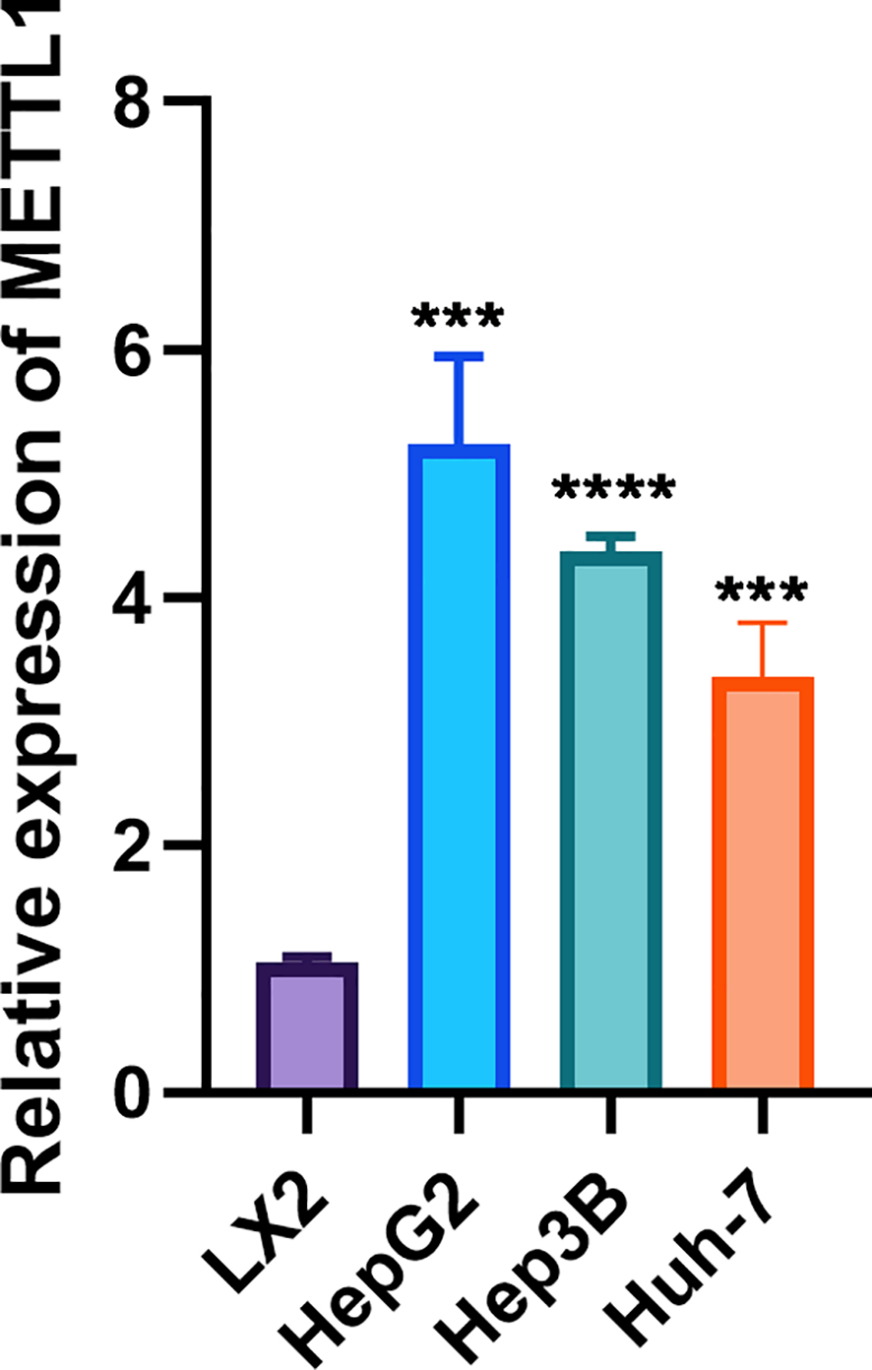
RT-qPCR showed that METTL1 was highly expressed in hepatocellular carcinoma cells. In the figure, ***, **** denote statistical significance levels indicating p < 0.001 and p < 0.0001, respectively.

Given the role of METTL1 across multiple analyses, we sought to validate its expression *in vitro* using cell lines. By RT-qPCR, we found that METTL1 mRNA was significantly upregulated in hepatocellular carcinoma cells compared to normal liver cells (p < 0.001, **Figure 10**). Notably, our pan-cancer analysis revealed a significant positive correlation between METTL1 expression and the levels of immune checkpoints such as PD-L1 (CD274) and CTLA-4 in HCC. In the TCGA-LIHC cohort, patients with high METTL1 expression also exhibited elevated expression of these inhibitory molecules, suggesting that METTL1 may drive immune evasion by upregulating immune checkpoints. These findings indicate that METTL1-mediated modulation of checkpoint expression plays a critical role in shaping the tumor immune microenvironment.

## 4 Discussion

HCC remains a formidable clinical challenge, with low survival rates and limited efficacy of current treatments such as immune checkpoint inhibitors. Although the risk factors for HCC have been well documented, these persistent clinical challenges underscore the urgent need for novel therapeutic targets. Given the critical role of nucleotide metabolism in both tumor progression and immune modulation, our study focused on nucleotide metabolism-related genes as a foundation for constructing a prognostic model. Our study addresses a significant knowledge gap by demonstrating that reprogramming of nucleotide metabolism—driven by METTL1 overexpression—not only supports tumor growth but also plays a pivotal role in HCC immune evasion. While previous research has primarily focused on general metabolic dysregulation in HCC, the specific mechanisms linking nucleotide metabolism to immune checkpoint regulation have remained unclear. Our data indicate that METTL1 upregulates key immune inhibitory molecules, such as PD-L1 and CTLA-4, thereby contributing to an immunosuppressive microenvironment and facilitating immune escape. These findings highlight the previously unexplored role of METTL1 in coordinating metabolic and immune regulatory networks and underscore its potential as a novel therapeutic target to reverse immune suppression in HCC.

Our molecular typing revealed two distinct HCC subgroups (C1 and C2), with C1 displaying lower overall metabolic activity, older patient age, and more advanced disease—a pattern that underscores the link between altered metabolism and worse clinical outcomes. This subgroup also showed downregulated pathways related to ribosome synthesis, cell proliferation, and immune response, while pathways involved in lipid and amino acid metabolism were upregulated, highlighting the complex interplay between metabolic adaptations and tumor behavior. By leveraging WGCNA, we identified key gene modules closely tied to nucleotide metabolism and prognosis, which aligns with growing evidence that dysregulated metabolic pathways can drive aggressive phenotypes in HCC. The successful application of multiple machine learning algorithms to derive a robust prognostic signature—validated across four independent cohorts—further indicates the clinical relevance of our approach. These findings not only emphasize the value of combining transcriptomic clustering with network analysis but also suggest that defining tumor subtypes by metabolic and immunological features may enhance risk stratification and inform targeted therapeutic strategies in HCC.

We analyzed and compared the diversity of expression profiles between risk groups. By analyzing Sankey’s chart, we found that the low-risk group was mainly distributed in C2 and survival samples, while the high-risk group was mainly distributed in C1 and had a worse prognosis, which was consistent with the results of the previous study. Subsequently, we performed differential expression analysis and found that RAD54L, MYBL2, CDC20 and other genes were significantly overexpressed in the high-risk group. Among them, RAD54L is a member of the SWI2/SNF2 chromatin remodeling protein family (18, 19), which is involved in promoting the development of various tumor types (20). MYBL2 (alias B-Myb) is a transcription factor in the MYB family of transcription factors that is a physiological regulator of cell cycle progression, cell survival, and cell differentiation (21). MYBL2 is also overexpressed in many cancer entities and associated with poor prognosis (22). Cell Division cycle protein 20 (Cdc20) is a member of the cyclin family (23), overexpressed in various cancer stem cells and malignant tumors such as hepatocellular carcinoma (24), may affect cell growth and tumorigenesis when its function is abnormal (25, 26). The high-risk group had lower activity in classical cancer-related pathways and higher expression levels of model genes. In the high-risk group, TP53 had the highest mutation frequency, and in the low-risk group, CTNNB2 and TTN had the highest mutation frequency, both of which were dominated by missense mutations. Risk scores were positively associated with most of the 14 classical cancer-related pathways, with the strongest positive correlation with Hypoxia.

Then, our immune microenvironment analysis suggests that high-risk tumors harbor greater infiltration of immunosuppressive cell subsets (e.g., Tregs, exhausted CD8^+ T cells), potentially explaining their poorer outcomes and reduced responsiveness to immunotherapies. Single-cell profiling provides further insight by demonstrating that non-responder samples exhibit elevated signature scores associated with aggressive tumor phenotypes, as well as enrichment of pathways implicated in immunosuppression and diminished immune recognition. These observations underscore a critical interplay between the tumor’s intrinsic molecular alterations and its extrinsic immune landscape—both of which converge to foster an environment conducive to immune escape. Consequently, our findings highlight the need for therapeutic strategies that not only target oncogenic drivers but also address the complex immunosuppressive mechanisms at play in high-risk HCC.

Finally, we analyzed the effects of METTL1 on the immune landscape. Methyltransferase-like protein-1 (METTL1) is a component of the m7G tRNA methyltransferase complex (27). The expression level of METTL1 is significantly up-regulated in HCC and negatively correlated with the survival rate of HCC patients. Mettl1 can also promote the development and progression of HCC by mediating m7G tRNA modification (28). Therefore, METTL1 and m7G modifications can be used as biomarkers or potential intervention targets to participate in improving early diagnosis and treatment of tumors (29). In addition, the METTL1-TGF-β2-PMN-MDSC axis may help restore anti-tumor immunity and prevent HCC recurrence after radiofrequency (RFA) treatment (30). Pan-cancer analyses highlight METTL1’s immunomodulatory impact, especially in HCC, where it correlates with checkpoint molecules (e.g., PDCD1, CTLA4) and immunoregulatory factors—potentially fostering immune escape. *In vitro* assays confirm METTL1 upregulation in hepatocellular carcinoma cells, underscoring its oncogenic and immunosuppressive roles. These observations, in line with broader evidence linking m7G modifications to tumor progression, suggest that METTL1 may serve as both a prognostic marker and a therapeutic target. Inhibiting METTL1 activity or detecting METTL1-related RNAs in peripheral blood could offer promising strategies to limit tumor growth and enhance immunotherapy efficacy in HCC.

In addition to driving nucleotide metabolism reprogramming, our study shows that METTL1 significantly influences the tumor immune microenvironment by regulating key immune checkpoints, such as PD-L1 and CTLA-4. The observed positive correlation between METTL1 and these checkpoints suggests a mechanism by which METTL1 contributes to immune evasion in HCC. By upregulating inhibitory checkpoint molecules, METTL1 may diminish the activation and infiltration of cytotoxic T cells, thereby facilitating tumor progression and resistance to immunotherapy. This dual role not only reinforces the prognostic value of METTL1 but also supports its potential as a therapeutic target. Targeting METTL1 could simultaneously disrupt aberrant metabolic pathways and reverse immune suppression, paving the way for more effective combination therapies in HCC.

Recent studies have not only highlighted the oncogenic role of METTL1 in hepatocellular carcinoma (HCC) but have also drawn attention to other components of the m7G-modifying complex. For instance, WDR4—the obligate binding partner of METTL1—has been reported to be overexpressed in HCC and contributes to tumor progression through its role in m7G tRNA methylation (31). Although both METTL1 and WDR4 are essential for catalyzing m7G modifications, emerging evidence suggests that their individual contributions might differ. Our multi-omics analysis reveals that METTL1 overexpression not only reprograms nucleotide metabolism but also significantly remodels the tumor immune microenvironment via upregulation of key immune checkpoints such as PD-L1 and CTLA-4. This dual functionality underscores a potentially more central or distinct role of METTL1 compared to its partner enzymes. In contrast, while studies on WDR4 predominantly focus on its role in promoting tumor cell proliferation and protein translation efficiency, the present study provides new insights into how aberrant m7G modification—mediated primarily by METTL1—can drive immune evasion. Such differences highlight the necessity to further dissect the individual and collaborative roles of m7G-modifying enzymes in HCC, which may ultimately aid in the development of targeted therapeutic strategies aimed at disrupting these epitranscriptomic regulatory networks.

Building on comprehensive multi-omics analyses similar to those used to delineate NUP62’s role in cancer progression and immune regulation (32), our findings underscore the translational potential of METTL1 as a therapeutic target in HCC. Beyond its prognostic significance, our findings underscore the translational potential of METTL1 as a therapeutic target. Future studies should investigate METTL1 inhibitors in preclinical HCC models, both as monotherapy and in combination with immune checkpoint blockade (e.g., anti-PD-1/PD-L1 or anti-CTLA-4 antibodies). *In vitro* experiments could assess whether METTL1 inhibition reduces immune checkpoint expression and enhances T cell activation, while *in vivo* studies could evaluate the effects on tumor growth and immune cell infiltration. Such combination strategies may synergistically disrupt aberrant nucleotide metabolism and reverse immune suppression, ultimately improving therapeutic outcomes in HCC.

From a translational perspective, inhibiting METTL1 could hold therapeutic promise, as it may sensitize tumors to immunotherapies by reducing immune checkpoint expression and reversing immunosuppression. Recent integrative studies, including disulfidptosis‐based risk assessments in glioma and analyses of the biophysical properties of cancer cells, underscore that metabolic regulators critically influence both the tumor microenvironment and immune evasion mechanisms (33). In light of these findings, future studies should investigate METTL1 inhibitors—alone and in combination with immune checkpoint blockade (e.g., anti-PD-1/PD-L1 or anti-CTLA-4 antibodies)—in preclinical HCC models. In addition, complementary strategies that harness the unique electrical, optical, and magnetic characteristics of cancer cells have been shown to improve targeted drug delivery and may further potentiate the effects of METTL1 inhibition (34). Such combinations may prove synergistic, given that metabolic reprogramming and immune checkpoint pathways often converge to promote tumor growth. Moreover, emerging evidence on targeting the immune privilege of tumor-initiating cells suggests that disrupting these protective niches can enhance the overall efficacy of immunotherapies (35). Our study highlights the potential for detecting METTL1 or related biomarkers in peripheral blood, which could enable non-invasive monitoring of treatment responses or early recurrence. This aligns with recent findings that circulating biomarkers reflecting metabolic and immunological alterations provide dynamic insights into tumor behavior and therapeutic responsiveness (34).

In summary, our findings not only confirm the significant influence of METTL1 overexpression on HCC progression but also provide detailed mechanistic insights into how it orchestrates both metabolic and immune dysregulation. By placing these observations within the broader context of m7G modifications and immuno-oncology, we offer a foundation for future research directed at reversing METTL1-mediated immune suppression and improving therapeutic efficacy in HCC. This integrated view emphasizes the importance of targeting epitranscriptomic regulators in the quest for effective, personalized treatment strategies against this formidable disease.

## 5 Conclusion

In this study, we reveal for the first time that METTL1 is a key driver of nucleotide metabolism reprogramming in HCC. Our multi-omics analyses demonstrate that METTL1 overexpression is not only associated with enhanced nucleotide synthesis but also correlates with significant changes in the immune microenvironment, including altered immune cell infiltration and checkpoint expression. These findings suggest that METTL1 may contribute to immune evasion, providing a dual mechanism that supports tumor progression. Compared to previous studies that have focused on general metabolic alterations, our work highlights the innovative concept that targeting METTL1 could simultaneously disrupt tumor metabolism and reverse immunosuppression. This novel insight lays the groundwork for future therapeutic strategies aimed at METTL1 inhibition to restore anti-tumor immunity and improve patient outcomes. To clarify the link between METTL1-mediated nucleotide metabolism and the HCC immune microenvironment, it is essential to establish that METTL1-driven RNA modifications directly modulate immune responses, elucidate how METTL1 regulates key checkpoints such as PD-L1 and CTLA-4, assess whether targeting METTL1 and its associated pathways can reverse immune evasion and enhance immunotherapy efficacy, and determine how changes in nucleotide metabolism affect the recruitment and function of immune cell subsets. Addressing these points will deepen our understanding of HCC pathogenesis and support the development of novel, personalized therapeutic strategies based on METTL1 inhibition.

## Data Availability

The data presented in the study are deposited in the TCGA repository (accession number TCGA-LIHC), GEO repository (accession numbers GSE14520 and GSE76427), and ICGC repository (accession number ICGC-JP). The metabolism-related gene sets were obtained from the MsigDB repository (https://www.gsea-msigdb.org/gsea/).
